# Coresidence of Older Parents and Adult Children Increases Older Adults' Self-Reported Psychological Well-Being

**DOI:** 10.1155/2022/5406196

**Published:** 2022-01-27

**Authors:** Soohyoung Rain Lee, Laurie S. Kim

**Affiliations:** ^1^Yeshiva University, Wurzweiler School of Social Work, USA; ^2^Department of Biology, University of Nevada, Las Vegas, USA

## Abstract

A multigenerational household is no longer a rare phenomenon in contemporary society. However, relevant literature has focused on elderly parents receiving support from their adult child, thereby coresiding. This is potentially problematic, as both generations could benefit from living together, and little is known about the benefit of living with adult children from older adults' perspectives compared to the risk of this living situation. Previous research suggests a significant negative effect of living alone, e.g., low psychological well-being, and it becomes more salient among single parents, such as widowed or divorced. The current paper utilizes the National Health Measurement Study with a sample of age 55 and over. Their SF-36 Mental Health and Physical Health Component and self-acceptance scores were measured. Path analysis reveals that both physical and mental health and self-acceptance scores are lower among single older adults at the time of the survey (e.g., divorced and widowed) than among those who are nonsingle and living with their adult child. A complete mediation effect of living with an adult child on older adults' mental health and self-acceptance was observed in both White and non-White minority older adults. This suggests that living with an adult child possibly serves as a protective factor for the negative relationship between living alone and their well-being. The current study seeks to stimulate ideas that might generate the following answer to community-based care in our contemporary aging society.

## 1. Introduction

Coresidence of older parents and adult children is no longer a rare phenomenon in the United States since the great recession. Recent census data suggested that about 30 percent of households now live with their adult child or are multigenerational households [1]. However, living arrangement literature has primarily focused on elderly parents living with their adult children to receive support and care due to limited physical and cognitive abilities. Therefore, recent studies have examined children caregivers' well-being [2]. Even though living with elderly parents might result in adverse outcomes from a child's view, multigenerational households possibly have positive effects. Importantly, little is known about how coresiding with adult child impacts elderly parents' well-being. This study was aimed at investigating the coresidence of adult children and elderly parents which possibly serves as a protective factor to the adverse outcomes of living alone among elderly parents.

### 1.1. Living Arrangement

Living arrangement literature has focused on understanding adult children taking care of their older parents with mental and physical deficits, such as dementia and chronic diseases. For example, elderly parents are likely to expect their children to take care of them when it is needed, and children also believe that taking care of elderly parents is one of their responsibilities [3, 4]. However, older adults' physical and cognitive health is involuntary circumstances where they are likely to become primary caregivers. Several factors may influence the recent pattern of coresidence of adult children and their older parents.

Two-generation coresidence is more common in Asian counties (e.g., eastern culture). Eastern culture includes Confucian ideals of filial piety, which indicates taking care of their elderly parent. Therefore, it is more common for Asian communities to live with their older parents than for a Westernized household [5]. Interestingly, one study reported no significant differences in coresidence of adult children and elderly parents by cultural and ethnic backgrounds [6]. In other words, residing with elderly parents may be caused by factors other than simply cultural differences.

Unlike cultural differences and child responsibilities of taking care of their older parents, individual older adults prefer to live independently rather than live with their adult child. A recent survey by Hannon shows that more than half of senior participants stated that they would prefer to live independently rather than reside with their adult children [7]. Coresidence of elderly parents and their adult children becomes an inevitable situation under various circumstances. For instance, family structures are strongly related to one's socioeconomic status [8]. In other words, older adults with mid-to-low income tend to live with their adult children compared to financially stable seniors. As a consequence of the increased housing market price, living with older parents has increased despite one's will [9, 10].

Unmarried adult children tend to move in with their adult parents compared to married ones, and notably, daughters are more likely to coreside with their parents than sons. Mothers are more likely to live with their children than fathers [3, 4]. Widowed, divorced, or single parents are more likely to live with their adult children than parents with significant others, married, remarried, or cohabitating with a boyfriend or girlfriend [5]. Coresidence of adult children and elderly parents can be due to involuntary circumstances (e.g., parents' illness).

### 1.2. The Benefit of Coresidence of Adult Child and Elderly Parent

Even though studies have focused on adult child's views and attitudes on living with their elderly parents, those studies are aimed at understanding the caregiving burden [11]. Two-generation coresidence can result in positive outcomes for both. Individual older adults reported higher psychological well-being when they lived with two or more generations compared to the single generation (live independently or without child or grandchild) [12, 13]. However, both studies were examined in China, and little is known how this coresidence may impact the American population [14].

As coresidence of adult children and older parents is no longer a rare phenomenon in contemporary societies and can be associated with one's later life satisfaction, it is important to explore this living structure's positive and negative effects.

## 2. Methods

The data for the study was collected from the United States National Health Measurement Study (NHMS; Fryback, 2012). NHMS surveyed 3844 (1641 males and 2203 females, and within this sample, 1086 were African American adults) in the continental United States to assess their physical function, mental function, social function, and other symptoms, as well as to understand their lifestyles and living situations. NHMS surveyed data that was publicly available through ICPRS.

### 2.1. Participants

“Old” has not been clearly defined with gradual changes in psychical and chronicle undergrowth in life expectancy. United Nations has not defined cut-off ages, but they agreed for the age range between 60 and 65 as older adults, and the World Health Organization (WHO) accepted the age of 55 as older adults for their analysis of Africa [15]. As age-related physical changes include risks of chronic diseases at ages of 50 and over (Hall et al., 2016), the “older adults” were considered 55 years old and over in this study.

### 2.2. Variables

#### 2.2.1. Older Adults' Characteristics

The older adults' race was recorded in a dummy variable (e.g., White = 0 and Hispanic/non‐White = 1).

#### 2.2.2. Living Situation

Samples were divided into two groups: coresiding with their adult children or living alone (0 = alone; 1 = coresiding with their adult children).

#### 2.2.3. Self-Reported Health

NHMS used SF-36 and systematically calculated the total score of mental health and physical health. Within the mental health component score, vitality (VT; e.g., full of life, energy, worn-out, and tired), social functioning (SF; e.g., social extent and social time), role-emotional (RE; e.g., cut down time, accomplished less, and less careful), and mental health (MH; e.g., nervous, down in dumps, peaceful, depressed/downhearted, and happy) scores are included [16]. Within the physical health component score, physical functioning (PF; e.g., vigorous activities, moderate activities, walk, and bathes), role-physical (RP; e.g., accomplished less and having difficulties), bodily pain (BP; e.g., pain magnitude and pain interference), and general health (GH; e.g., becoming sick easier and health getting worse) are included [16].

#### 2.2.4. Self-Acceptance

To measure positive attitude toward self and the levels of acceptances of self, including good and bad qualities, Ryff's psychological well-being scale was used (e.g., “When I look at the story of my life, I am pleased with how things have turned out” and “I like most aspects of my personality”) [17, 18].

### 2.3. Data Analyses

The Statistical Package for the Social Sciences (SPSS) version 25 analyzed demographic information and accounted for missing data. Mplus8 [19] was used to perform path analysis to determine the possible protective factors of coresidence of adult children and elderly parents. Within this analysis, 90% bias-corrected confidence intervals are based on 10000 [20]. A model fit was reported throughout.

## 3. Results

### 3.1. Samples

As shown in Table 1, of the 3844 initial samples, 2375 were identified as aged 55 and over (*M* = 69.51, SD = 8.55, maximum = 89). Of the final sample size of 2375, there was slightly more male (56.9%), 54.4% were single at the time of the survey (e.g., widowed and divorced), 71.1% were reported as White, and 28% were non-White/Hispanic/Black or African American. Less than 5% were Asian and others (e.g., Native American and Native Hawaiian). Moreover, more than half of the respondents have lived alone (82.6%) compared to those living with their children (17.4%).

### 3.2. Path Analysis: The Partial Mediation Effect from the Whole Sample

**Figure 1:**
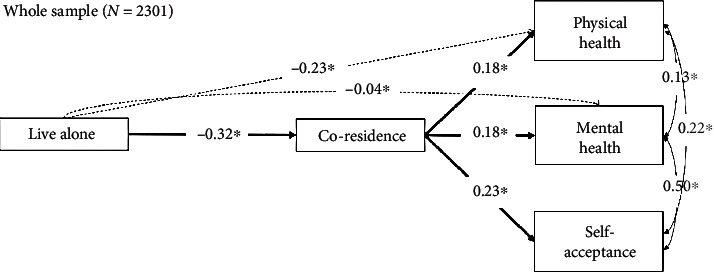
Whole sample path analysis.

Overall, path analysis with excellent goodness of fit (CFI = 0.99, TLI = 0.99, SRMR = 0.01). As shown in Figure 1, the whole sample path analysis revealed that single parents at the time of the survey (e.g., widowed and divorced) were less likely to coreside with their adult children (STDYX = −0.32; 95% CI [-0.36, -0.29], *p* ≤ 0.01). Living alone is negatively associated with physical health (STDYX = −0.23; 95% CI [-0.26, -0.19], *p* < 0.01), and single older adults were likely to report having a low mental health score (STDYX = −0.04; 95% CI [-0.08, -0.007], *p* < 0.05). Contrarily, older parents living with their children scored significantly higher for mental health (STDYX = 0.18; 95% CI [0.15, 0.22], *p* < 0.01) and scored significantly higher for self-acceptance level (STDYX = 0.23; 95% CI [0.19, 0.27], *p* < 0.01). There are partial mediation effect of living with their adult children and physical health and mental health. The negative association remained significant, when coresiding with their adult children (STDYX = −0.23; 95% CI [-0.26, -0.19], *p* = 0.01 and STDYX = −0.04; 95% CI [-0.081, -0.007], *p* < 0.05, respectively), whereas coresidence status mediated the negative association between living alone and self-acceptance (refer to Table 2).

### 3.3. Path Analysis: Grouping by Race

**Figure 2:**
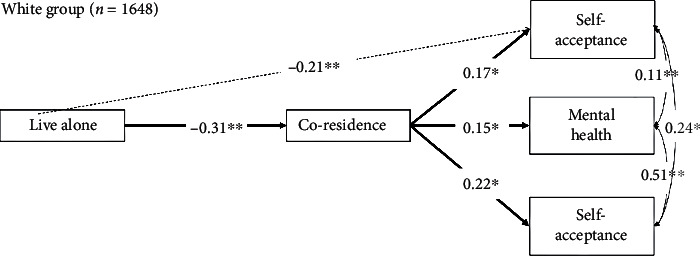
Subgroup path analysis (White group, *n* = 1648).

**Figure 3:**
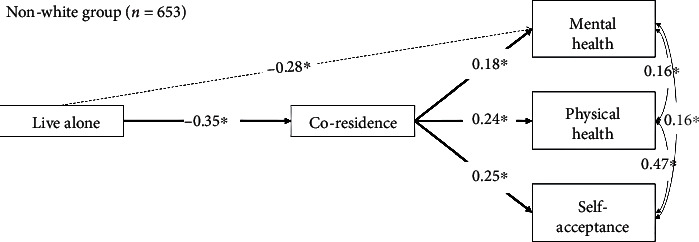
Subgroup path analysis continuation (non-White group, *n* = 653).

The same analyses were performed with two different groups. As seen in Figures 2 and 3, White and non-White individuals showed similarity to the whole group; within the White group, single older adults are less likely to live with their adult children (STDYX = −0.31; 95% CI [-0.36, -0.26], *p* ≤ 0.01) and reported having low level of physical health (STDYX = −0.21; 95% CI [-0.25, -0.17], *p* < 0.01). Coresiding with their adult children was positively associated with physical health (STDYX = 0.17; 95% CI [0.12, 0.21], *p* < 0.01) and mental health (STDYX = 0.15; 95% CI [0.10, 0.19], *p* < 0.01) and the levels of self-acceptance (STDYX = 0.25; 95% CI [0.18, 0.27], *p* < 0.01). The results are synchronized with both white and non-White groups.

## 4. Discussion

Since the early 2000s, the proportion of multigeneration households has been increased [9, 21], and the social norms about coresiding with adult children from older adults' perspectives might have changed. The current study suggested that coresiding with adult children may decrease the risks of living alone for single older parents. As a result, both physical health and mental health were negatively associated with being alone. On the contrary, individuals living with their adult children were likely to report having good physical and mental health. In the current study, mental health was measured by individuals' levels of social functioning and happiness [16, 22]. This coincides with positive effects of multigenerational households where older parents continue to be the provider for their adult child [23] and report alleviation in loneliness [24, 25], and also, the changes in the role of parents to grandparents increase older parents' psychological and physiological well-being [13].

Importantly, a complete mediation effect was reported in both White and non-White individual older single parents. This suggests that regardless of older adults' social and cultural background, living with their children may be a protective factor in the negative consequences of being alone. This is an important finding as self-acceptance is one of the keys to well-being in older adults [26], and the results argue with the negative aspect of coresiding with an adult child, such as their child's quality of marriage [27] and possibly the decrease in older adults' mental health and psychological well-being due to additional burden by supporting their adult children [28].

Current findings suggest the double-edge sword outcomes of coresidence of adult children and older adults. On the one hand, multigeneration coresidence benefits older adults with physical and mental health and self-acceptance. On the other hand, the older individuals' physical health remained negatively associated with being alone even when coresiding with an adult child was adjusted. This indicates that individuals with low physical health may not be welcomed in adult children's homes due to limited physical activity capacity, thereby depending on their child for daily activities. Single parents at the interview were less likely to live with their adult children and reported having a lower level of physical and mental health which is another concern since older adults' limited physical health and the level of dependency are two of the core factors for older individuals to be admitted to the nursing facility [29].

Despite its meaningful findings, this study is not without limitations. One of the severe limitations is that the data was collected in 2006-2007. It is relatively outdated, and the trend of living arrangements has possibly changed over time since the past decades. As aforementioned, the most recent census suggested the increase in coresidence of older parents and adult children [9, 21], and both benefits and harms have been studied in recent years, but primary samples were either Europe or Asia [24, 30]. Further, recent studies that were attempted to understand individuals' psychosocial well-being used the same dataset, which suggests that there has not been much attention regarding coresidence among the U.S. population [31, 32]. Also, the current study is not aimed at discussing the trend of living arrangements for older adults and their children. The aim was to understand how coresiding with adult children may impact older parents' psychosocial well-being. The interview year would be less influential in this case, and further study with a newer dataset is recommended.

Even though older adults are likely to move in with their adult children to receive such support when they lack physical and cognitive abilities to be independent, the current study included healthy older adults. On the one hand, there are possible exchanges in support, including instrumental and material support, which can enhance the quality of living and life for both generations [23]. On the other hand, these positive outcomes may not appear with comparably unhealthy older adults. Further research is recommended to investigate the different effects on older adults' health status.

## 5. Conclusion

Culturally, two or more generations living in the same household are not as common as in other eastern cultures. The coresidence of adult children and elderly parents is no longer a rare phenomenon in the United States. Assumedly, older adults live with their adult children to receive needed care and support when lacking independence due to health-related problems. Previous studies have suggested that both adult children and older adults coreside due to financial reasons, including employment and housing crisis. The patterns of living arrangements have changed since 2008 [9], and the current study suggested that older adults living with their adult children can be a protective factor for the negative consequences of being alone among those older parents. The benefit of multigeneration is recommended to be considered when providing support for those single, living alone older individuals.

## Figures and Tables

**Table 1:** Demographic information for the sample (*N* = 2376)^1,2^.

	*M*	Range	*n*	(%)
Age	77.82	97		
Gender				
Male			1351	56.9%
Female			1025	43.1%
Race^3^				
White			1660	70.1%
Black or African-American			618	26.1%
Others			90	3.8%
Minorities				
White			1689	71.6%
Non-White			671	28.4%
Single status				
Yes			1292	54.4%
No			1083	45.6%
Living arrangement				
Living alone			1961	82.6%
Living with kids			412	17.4%

^1^Valid percentages are reported. ^2^Missingness is excluded. ^3^White and non-White were used in analyses.

**Table 2:** Results of path analysis (*N* = 2376).

	*B*	95% CI	STDYX	95% CI
Living alone ⟶ mental health	-2.14^∗^	-2.89, -1.38	-0.10^∗^	-0.14, -0.06
Living alone ⟶ physical health	-5.52	-6.39, -4.65	-0.23	-0.26, -0.19
Living alone ⟶ self-acceptance	-4.19^∗^	-5.78, -2.59	-0.10^∗^	-0.14, -0.06
Coresidence ⟶ mental health	1.28^∗^	1.04, 1.52	0.20^∗^	0.16, 0.23
Coresidence ⟶ physical health	1.35	1.07, 1.64	0.18	0.14, 0.22
Coresidence ⟶ self-acceptance	3.05^∗^	2.59, 3.52	0.24^∗^	0.21, 0.28
Living alone ⟶ coresidence ⟶ mental health	-1.26^∗∗^	-1.51, -1.10	-0.06^∗∗^	-0.07, -0.05
Living alone ⟶ coresidence ⟶ physical health	-1.43^∗∗^	-1.73, -1.14	-0.06^∗∗^	-0.07, -0.04
Living alone ⟶ coresidence ⟶ self-acceptance	-3.12^∗∗^	-3.67, -2.57	-0.07^∗∗^	-0.09, -0.06

^∗^ *p* ≤ 0.05.

## Data Availability

The authors have used publicly available data.
